# Specificity of the chromatic noise influence on the luminance contrast discrimination to the color vision phenotype

**DOI:** 10.1038/s41598-020-74875-3

**Published:** 2020-10-21

**Authors:** Bruna Rafaela Silva Sousa, Terezinha Medeiros Gonçalves Loureiro, Paulo Roney Kilpp Goulart, Maria Izabel Tentes Cortes, Marcelo Fernandes Costa, Daniela Maria Oliveira Bonci, Luiz Claudio Portnoi Baran, Einat Hauzman, Dora Fix Ventura, Leticia Miquilini, Givago Silva Souza

**Affiliations:** 1 Núcleo de Medicina Tropical, Universidade Federal do Pará, Av. Generalíssimo Deodoro 92, Umarizal, Belém, Pará 66055-240 Brazil; 2 Instituto de Ciências Biológicas, Universidade Federal do Pará, Belém, Brazil; 3 Núcleo de Teoria e Pesquisa do Comportamento, Universidade Federal do Pará, Belém, Brazil; 4 Centro de Ciências da Saúde, Universidade Federal do Amapá, Macapá, Brazil; 5 Instituto de Psicologia, Universidade de São Paulo, São Paulo, Brazil

**Keywords:** Sensory processing, Neuroscience, Visual system, Colour vision, Pattern vision

## Abstract

Many studies have examined how color and luminance information are processed in the visual system. It has been observed that chromatic noise masked luminance discrimination in trichromats and that luminance thresholds increased as a function of noise saturation. Here, we aimed to compare chromatic noise inhibition on the luminance thresholds of trichromats and subjects with severe deutan or protan losses. Twenty-two age-matched subjects were evaluated, 12 trichromats and 10 with congenital color vision impairment: 5 protanopes/protanomalous, and 5 deuteranopes/deuteranomalous. We used a mosaic of circles containing chromatic noise consisting of 8 chromaticities around protan, deutan, and tritan confusion lines. A subset of the circles differed in the remaining circles by the luminance arising from a C-shaped central target. All the participants were tested in 4 chromatic noise saturation conditions (0.04, 0.02, 0.01, 0.005 u′v′ units) and 1 condition without chromatic noise. We observed that trichromats had an increasing luminance threshold as a function of chromatic noise saturation under all chromatic noise conditions. The subjects with color vision deficiencies displayed no changes in the luminance threshold across the different chromatic noise saturations when the noise was composed of chromaticities close to their color confusion lines (protan and deutan chromatic noise). However, for tritan chromatic noise, they were found to have similar results to the trichromats. The use of chromatic noise masking on luminance threshold estimates could help to simultaneously examine the processing of luminance and color information. A comparison between luminance contrast discrimination obtained from no chromatic and high-saturated chromatic noise conditions could be initially undertaken in this double-duty test.

## Introduction

Color and luminance perceptual interactions have been described under different experimental conditions, where one visual information masks the perception of another^1–3^. Depending on the experimental design, these studies have found a bimodal influence of luminance contrast on color discrimination^2–4^. These studies reported that low-to-medium luminance contrast (up to 30 × threshold) facilitated chromatic discrimination, while high luminance contrast restrained chromatic discrimination. Conversely, color contrast masking has been characterized as an inhibitor of luminance discrimination^2,5^ or as having no effect on luminance contrast perception^3^.

Miquilini et al.^5^ introduced a method to investigate color masking in spatial luminance contrast discrimination. A chromatic noise was applied to a mosaic stimulus that had a target contrasting luminance in the background. They found that the luminance contrast threshold changed as a function of the saturation of the chromatic noise. The higher the chromatic noise saturation, the higher the luminance contrast threshold. This method was only applied in normal trichromats. Therefore, it is unclear how the luminance contrast thresholds of subjects with congenital color vision deficiency would be affected.

It is well established that human trichromatic color vision uses three cone photopigments with different spectral sensitivities to organize a three-dimensional perceptual color space (black-white, red-green, and blue-yellow axes)^6–9^. The absence of one or more photopigments changes this perceptual organization. Protanopes (lack of L-cone photopigment) and deuteranopes (lack M-cone photopigment) have dichromatic color vision with black-white and blue-yellow perceptual dimensions and no red-green axes. Tritanopes (lack S-cone photopigment) also have dichromatic color vision but with black-white and red-green perceptual dimensions and no blue-yellow channel^10^. We hypothesized that chromatic noise containing chromaticities that cannot be discriminated against by each type of colorblindness would either not influence or have a minor effect on luminance contrast discrimination.

We used three different stimuli with chromatic noise comprised of chromaticities located close to the protan, deutan, and tritan confusion lines in a perceptually homogeneous color diagram. The luminance contrast thresholds of trichromats and subjects with severe protan or deutan losses were compared under each chromatic noise condition.

## Methods

### Ethical considerations

This cross-sectional investigation was approved by the Ethical Committee of the Tropical Medicine Institute of the Federal University of Pará, Brazil (report# 2.207.434), in accordance with the Declaration of Helsinki of 1964 and any subsequent updates. We obtained written informed consent for their participation.

### Subjects

Twenty-two participants were recruited (13 males, 7 females, 25.15 ± 5.02 years). The participants had normal or corrected 20/20 visual acuity. Participants had no history of neurological or systemic disease that would affect their luminance or color vision.

### Experimental procedures: color vision phenotype evaluation

We used the Ishihara test and Color Assessment and Diagnosis test (CAD) to classify the phenotype of color vision deficiencies. We used the 38-plate full version of the Ishihara test, 1997 edition (Graham- Field Inc., Doraville, GA, USA). The plates were shown for 3 s from a 75 cm viewing distance under standard illumination. The subjects were instructed to read the number on the plate during the presentation. We evaluated the performance of the subjects by computing the number of errors in the test. Participants with errors ≥ 8 were genetically analyzed to identify the presence or absence of the X-linked L and M-opsin genes in the genomes.

The CAD test presents a chromatic square target moving on a grey background. The subject’s task consists of reporting the direction of the target’s movement. Both target and background are masked by a spatial–temporal random luminance noise which compensates for differences in the luminous efficiency function of different participants and eliminates border cues. The result is to allow the observer's responses to be based only on chromatic differences between the target and the stimulus background^11,12^. The CAD test was run using a microcomputer (Dell, Intel Core i3, Intel HD graphics) that drives a 22 inch color LCD (NEC Model, MultiSync P221w, Japan). The visual stimuli comprised an array of squares (15 × 15) covering an area of 3.11 squared degrees of visual angle. Each element of the array had 0.21 squared degrees and had its luminance value randomly changed at intervals between 50 and 80 ms in a range between 25.5 cd/m^2^ and 42.5 cd/m^2^. A subset of the array (target) had a different background chromaticity (CIE 1976: u′ = 0.1947; v′ = 0.4639). The target area was 1.04 squared degrees. The distance from the display to the observer was 1.4 m, and the observer’s task was to identify the direction of the target movement among 4 diagonal alternatives (up-right, up-left, bottom-right, and bottom-left). The stimulus presentation lasted for 2 s. The vector distance between the chromaticities of the target and background was controlled by a staircase procedure that stopped after 12 reversals. Sixteen chromatic axes (6 protan axes, 6 deutan axes, and 4 tritan axes) were used to find the chromatic discrimination thresholds. The average of the last 6 reversals estimated using the protan, deutan, and tritan stimuli were considered as the protan, deutan, and tritan thresholds. High thresholds indicated a reduction in chromatic discrimination in a chromatic axis. The CAD test returned a suggestion for the color vision phenotype of each participant based on the manufacturer’s normative database. This was used to characterize the participant’s phenotype.

### Experimental procedures: color vision genotyping

Participants whose Ishihara and CAD test results suggested color vision deficiency were genetically analyzed using the method proposed by Neitz and Neitz^13^. The presence or absence of the X-linked L and M visual pigment genes in the genome of the participants were determined, based on the amplification of exon 5 of both genes. This was followed by incubation with a specific restriction endonuclease that cleaves the amplified exon of one gene (L) but not the other (M). The test was not sensitive enough to determine the presence of multiple copies of one gene or the presence of hybrid genes. However, given that most of the spectral shift between the M and L opsins is generated by residues 277 and 285, located at exon 5, and that minor effects are caused by residue 180, at exon 3, it can be assumed that the presence of hybrid genes would generate visual pigments with minor shifts in their absorption peaks, and result in severe anomalous conditions in the color vision phenotype. Residues 277 and 285 are responsible for 10 and 17 nm shifts between M and L opsins, respectively, while residue 180 causes only 5 nm shifts^14^. Therefore, the genetic analysis performed in this study was used to classify the participants as normal trichromats or with severe congenital color vision impairments (either dichromats or severe anomalous trichromats), and to confirm the phenotypes observed in the color vision test.

**Figure 1:**
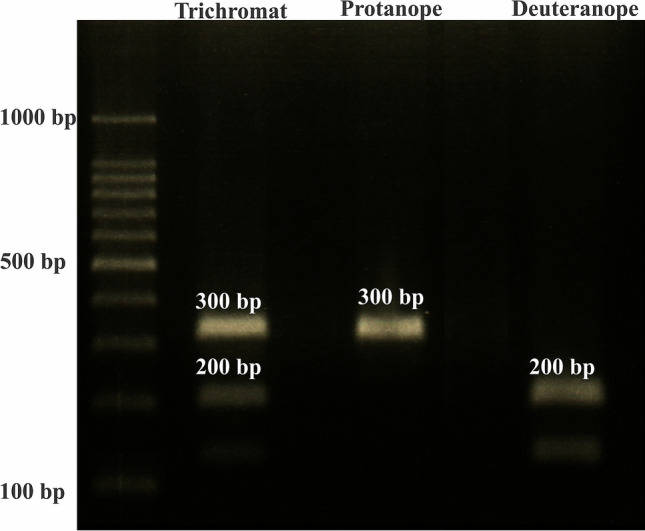
Agarose gel analysis with the amplified exon 5 fragments of the cleaved L-opsin gene (two lower bands) and the uncleaved M-opsin gene (higher band). Lane 1 shows a 100 bp ladder and numerical values indicate the number of base pairs; lane 2, a trichromat individual with both opsin genes; lane 3, a protanope individual, with no L-opsin gene; lane 4, a deuteranope individual, with no M-opsin gene. The sample lanes were from different gels and white spacing delineated the non-contiguous lanes.

For the genetic analyses, DNA samples were extracted from buccal brush and purified using the Gentra Puregene Buccal Cell Kit (Gentra Systems, Inc., Minneapolis, MN, USA), according to the manufacturer’s protocol. A fragment of approximately 300 bp, containing exon 5 of both L and M-opsin genes, was amplified by polymerase chain reactions (PCRs), using the primer pair described by Neitz and Neitz^13^. PCRs were carried out using High Fidelity Platinum Taq Polymerase, 10 × High Fidelity Buffer, MgCl_2_, 10 mM GeneAmp dNTPs (Applied Biosystems, Inc., Foster City, USA), and 20 mM primers in 50 µL reactions. PCR conditions included an initial DNA denaturation step at 94 °C for 1 min, 37 cycles of 94 °C for 15 s, annealing temperature at 57 °C for 30 s, and extension temperature at 72 °C for 30 s, followed by a final extension temperature of 72 °C for 7 min. The amplified fragments were then incubated with the restriction endonuclease Rsa I (Invitrogen, Carlsbad, USA), which cleaves exon 5 of the L-opsin gene, but not the M-opsin gene. The resulting PCR products were visualized using 1.0% agarose gel electrophoresis. Trichromat individuals were identified by the presence of three bands: a ~ 300 bp band from the uncleaved M-opsin fragment, and two smaller bands (with ~ 100 and ~ 200 bp) of the cleaved L-opsin gene fragment. Deuteranope and severe deuteranomalous individuals only display the two smaller L-opsin gene bands. Contrastingly, protanope and severe protanomalous individuals only display the larger 300 bp M-opsin gene band (Fig. 1). DNA samples from a known trichromat were used as controls.

### Color vision characterization

For all participants, their color vision phenotype was characterized using their performance on the CAD and Ishihara tests, while their color vision genotype was characterized by the presence of the exon 5 of the L and/or M-opsin genes in the genome. Participants with < 8 errors on the Ishihara test and the trichromat phenotype, as suggested by the CAD test, were classified as trichromat. Participants with ≥ 8 errors in the Ishihara test and a dichromat/severe anomalous trichromat phenotype, as suggested by the CAD test, were genetically confirmed as protanopes/protanomalous or deuteranopes/deuteranomalous, with the absence of intact L or M-opsin genes, respectively (Fig. 1).

### Experimental procedures: Luminance contrast discrimination masked by chromatic noise

We used a software programed in MATLAB (MATLAB 2017b, Mathworks, Natick, MA, USA) on a MacBook PRO platform (Apple Inc., Palo Alto, USA) with a panel built into the laptop (17″ liquid crystal display, 1680 × 1050 pixels of spatial resolution, frame rate of 75 Hz). A Macbook PRO drove an NVIDIA GeForce 8600M GT graphics processor with 512 MB of GDDR3 SDRAM and 10 bits of color resolution per channel. We used a chromameter (CS-100A, Konica Minolta, Osaka, Japan) to calibrate the display and all chromatic calculations in this study, assuming a 2° observer angle and a D65 illuminant. The stimulus was composed of a mosaic of 428 circles and 16 different mosaic arrangements that were randomly chosen. The diameter of the individual mosaic elements ranged between 0.12 and 0.49°.

Three protocols of chromatic noise were used: (i) protan chromatic noise, (ii) deutan chromatic noise, and (iii) tritan chromatic noise. Each noise was composed of 10 chromaticities that were projected radially from a reference chromaticity (CIE 1976: u′ = 0.1947; v′ = 0.4639). There were 5 chromaticities spaced 2 degrees apart from each side of each chromatic axis. Protan, deutan, and tritan chromatic axes used in the CAD test were used as a reference to choose chromaticities used in each noise condition. A protocol with no chromatic noise condition was also used as a control. In the no chromatic noise condition, there was a single chromaticity in the mosaic that was the reference chromaticity.

**Figure 2:**
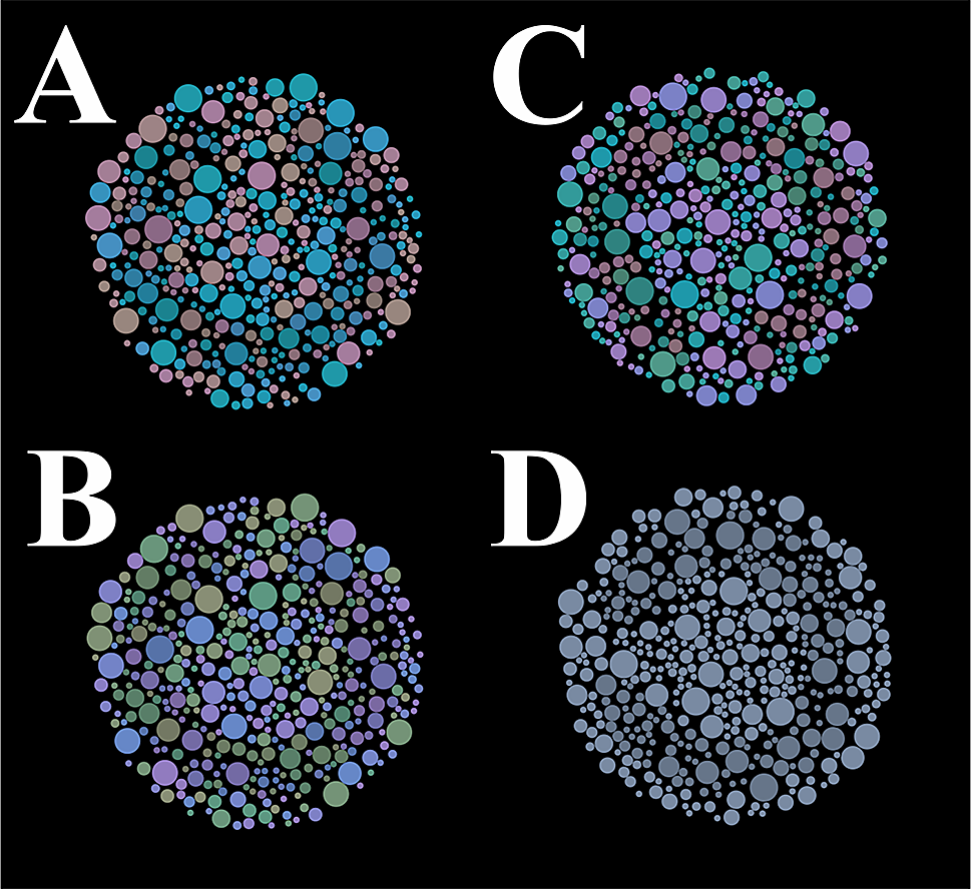
Stimuli used in experiments. The luminance contrast of the stimulus was represented by a C-shaped target. A chromatic noise was present in the stimulus to masking luminance contrast. Three conditions of noise were tested: (**A**) protan, (**B**) deutan, (**C**) tritan, and one condition with (**D**) no chromatic noise.

A subset of circles with luminance different from the background formed a Landolt’s C-shaped target. At the beginning of the test, the target luminance was 4 cd/m^2^ and the background luminance was 40 cd/m^2^. Figure 2 shows some examples of the stimulus for each chromatic noise.

For each chromatic noise protocol, 5 saturation conditions of the noise (vector size of 0.04, 0.02, 0.01, 0.005, and an isochromatic condition) were used to mask the luminance discrimination of the target. The observer had to indicate which orientation was Landolt’s C-gap (top, left, right, or bottom). The luminance discrimination task was performed using a two down/one up adaptive staircase procedure. This corresponded to 70.7% of correct responses. After 2 correct responses, the luminance of the target was increased. After 1 wrong response, the luminance of the target was decreased. The step size of the target luminance increase or decrease was calculated using Eq. (1). The target luminance (targetlum) of the trial (t) was summed (after 2 correct responses) or decreased (after one wrong response) by the difference between the background luminance (bglum) and the target luminance of the previous trial, times a factor (f). The factor values were 0.5 and 1.5 for the increase and decrease in the luminance step, respectively.

1 $$targetlum\left(t\right)={10}^{log10\left(targetlum\left(t-1\right)\right)\pm \left[log10\left(bglum\right)-log10\left(targetlum\right)\right]\times f}$$

A reversal was considered an incorrect response after 2 hits, or 2 hits following an incorrect choice. The staircase stopped after 12 reversals, and the averaged Weber contrast between the target and background of the last 6 reversals was recorded as the luminance contrast threshold.

### Data analysis

Luminance contrast thresholds $\left( \uppsi  \right)$ were normalized by the threshold obtained under the non-chromatic noise condition $({\uppsi}_{o})$. Luminance contrast thresholds, as a function of the chromatic noise vector, were fitted by Michaelis–Menten functions using the least-squares method (Eq. (2)). From each fit, the total change ($a$) in threshold and the semi-saturation constant $\left(\tau \right)$ was considered as an indicator of the gain of the mechanism.

2 $$\uppsi={\uppsi}_{o}+a\times \left(1-{e}^{\left(\frac{-x}{\tau }\right)}\right)$$

in which $\uppsi$ was the relative luminance contrast threshold in the chromatic noise vector size $x$, ${\uppsi}_{o}$ was the contrast threshold in the stimulus condition with no chromatic noise, $a$ represents the total change in the threshold, and $\tau$ is the semisaturation constant of the function.

We used a non-parametric Friedman test of differences among repeated measures followed by Dunn’s *post-hoc* test to compare the effect of chromatic noise vector size on luminance contrast thresholds, and on $a$ values estimated from the best-fitted functions. The Mann–Whitney U test was performed to compare the $a$ values obtained from trichromats and dichromats (protanopes and deuteranopes). For all statistical procedures, we considered the significance level to be 0.05 or corrected for multiple comparisons (α = 0.016).

## Results

**Figure 3:**
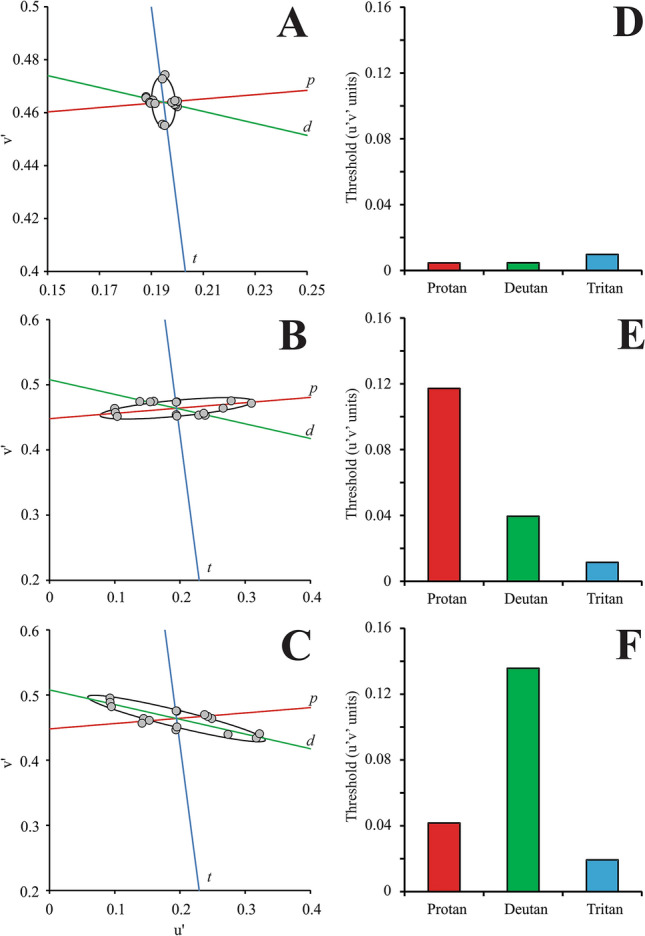
CAD test results. Color discrimination ellipses (black ellipse) from a trichromat subject (**A**), protanope subject (**B**), and deuteranope subject (**C**) and their respective mean threshold vector sizes for the protan (red bars), deutan (green bars), and tritan (blue bars) chromatic axes are represented in (**D**–**F**), respectively. The red, green and blue dashed lines represent the protan, deutan, and tritan color confusion lines.

For all subjects with color vision impairments, color vision genotyping was verified by phenotype evaluation using the color discrimination test. No participant was excluded based on their genetics. Figure 3 shows the color discrimination ellipses of a subject phenotypically characterized as a trichromat, and subjects phenotypically and genetically characterized as protanopes or severe protanomalous and as deuteranopes or severe deuteranomalous, respectively. Table 1 shows the vector thresholds obtained in the CAD test for all participants and their color vision characterization. The final analysis was conducted with 12 trichromats and 10 participants with severe protan (5) and deutan (5) deficits.

**Table 1:** CAD results and color vision characterization of the participants.

Participant	Threshold vector (u’v’ × 10^–4^)			Color vision characterization
	Protan	Deutan	Tritan	
T1	52.8	43.4	116.2	Trichromat
T2	46	44.4	102.2	Trichromat
T3	49.7	50.7	146.3	Trichromat
T4	58.9	67.6	164.8	Trichromat
T5	56.1	56	155.9	Trichromat
T6	23.9	20.4	30.1	Trichromat
T7	44.3	45.9	88.2	Trichromat
T8	59.1	53	185.9	Trichromat
T9	62.9	56.1	129.3	Trichromat
T10	56.1	56	155.9	Trichromat
T11	55.3	53.2	159.5	Trichromat
T12	47.5	52.2	155.4	Trichromat
P1	519	314	150	Protanope
P2	1228	539	186	Protanope
P3	1235	370	183	Protanope
P4	1451	1156	955	Protanope
P5	2058	1922	1948	Protanope
D1	643	1366	240	Deuteranope
D2	422	821	136	Deuteranope
D3	459	997	175	Deuteranope
D4	487	1279	198	Deuteranope
D5	676	1236	234	Deuteranope

### Comparison of the color discrimination thresholds for each color vision phenotype

For trichromats, we observed that the chromatic axis had a significant effect on the color discrimination vectors (H[2] = 16.95, p = 0.0002). The tritan thresholds were significantly higher than the deutan and protan thresholds (p < 0.05). There was no significant difference between the protan and deutan color discrimination thresholds.

For subjects with protan losses, we observed no significant effect of the chromatic axis on the color discrimination thresholds (H[2] = 3.44, p = 0.17). For those with deutan impairments, we observed a significant effect of the chromatic axis on the color discrimination thresholds (H[2] = 12.5, p = 0.0019). Deutan thresholds were significantly higher than the tritan thresholds (p < 0.05).

### Luminance contrast threshold as a function of the chromatic noise

**Figure 4:**
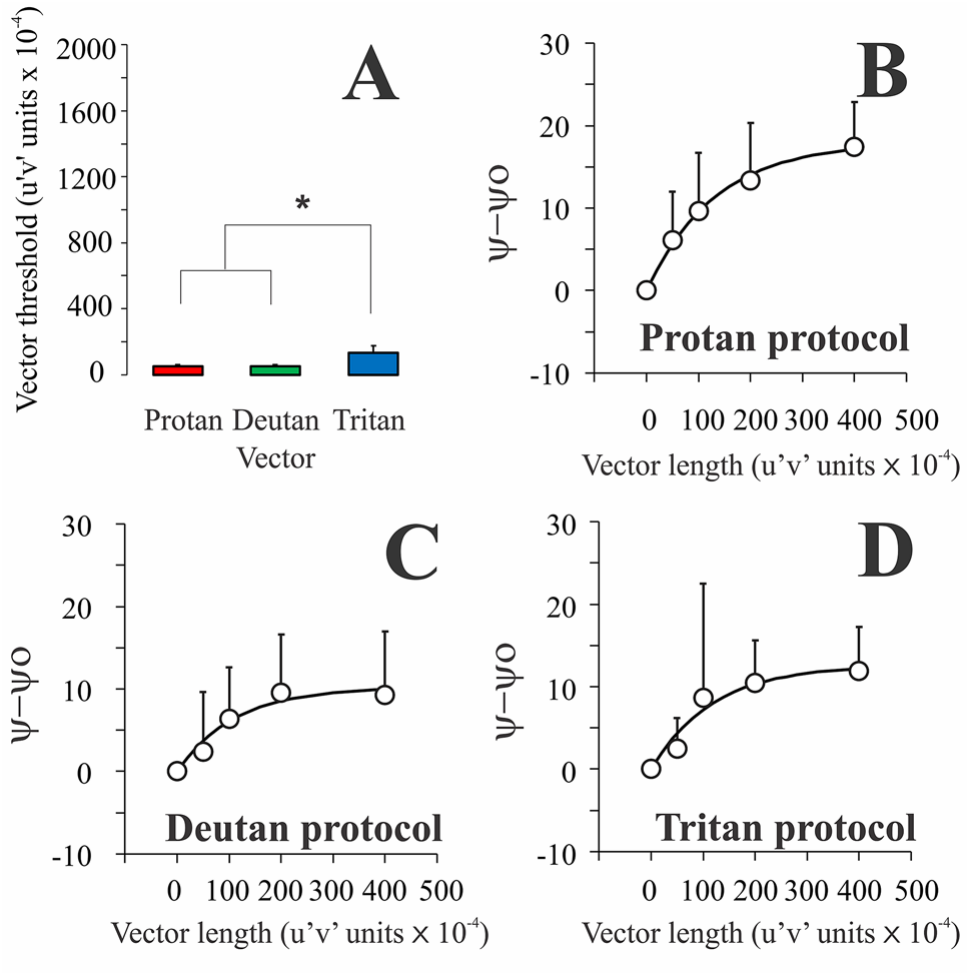
Trichromat group results. (**A**) Mean color discrimination thresholds estimated using CAD test for protan (red bar), deutan (green bar), and tritan (blue bar) confusion axes. (**B**) Mean luminance contrast threshold (black circles) as a function of the protan chromatic noise saturation. (**C**) Mean luminance contrast threshold (black circles) as a function of the deutan chromatic noise saturation. (**D**) Mean luminance contrast threshold (black circles) as a function of the tritan chromatic noise saturation. The black curve represents the best-fitted Michaelis–Menten function to the data. Error bars represent the standard deviation of the mean. Ѱ is the threshold estimated in a stimulus condition with chromatic noise, Ѱ_o_ is the threshold estimated in the stimulus condition with no chromatic noise.

**Figure 5:**
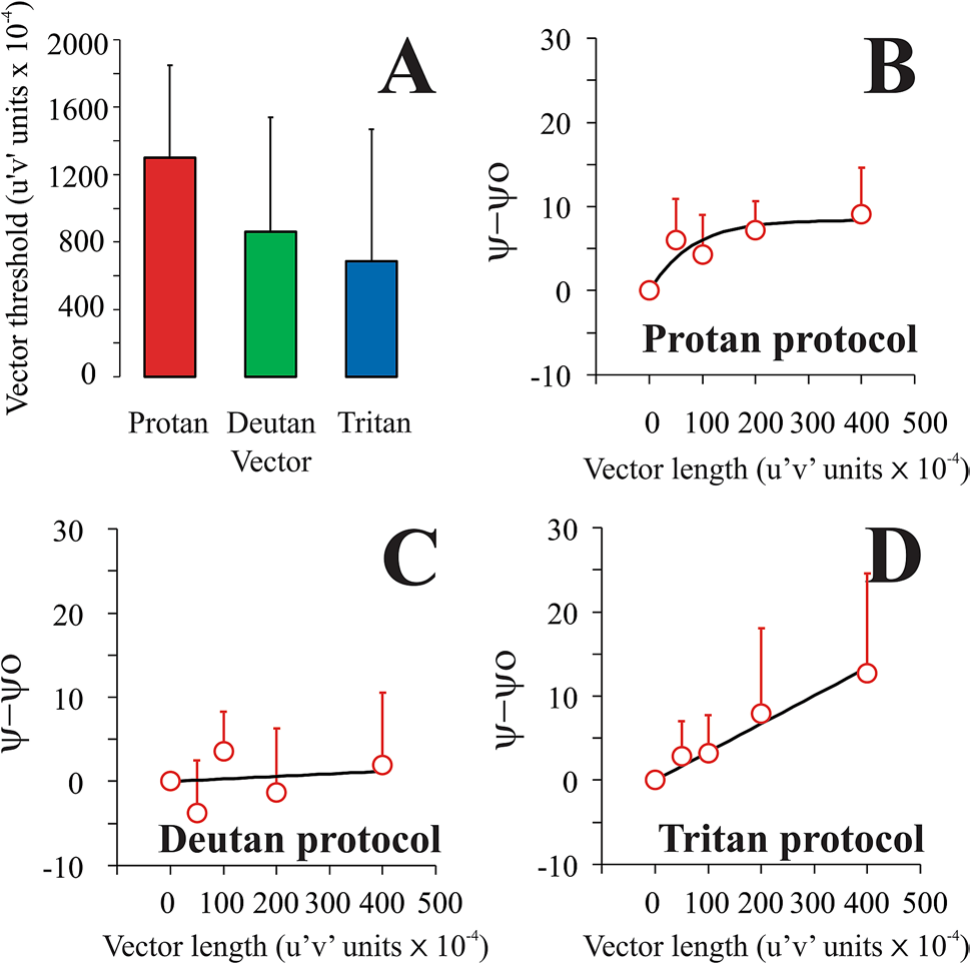
Protan group results. (**A**) Mean color discrimination thresholds estimated using CAD test for protan (red bar), deutan (green bar), and tritan (blue bar) confusion axes. (**B**) Mean luminance contrast threshold (red circles) as a function of the protan chromatic noise saturation. (**C**) Mean luminance contrast threshold (red circles) as a function of the deutan chromatic noise saturation. (**D**) Mean luminance contrast threshold (red circles) as a function of the tritan chromatic noise saturation. The black curve represents the best-fitted Michaelis–Menten function to the data. Error bars represent the standard deviation of the mean. Ѱ is the threshold estimated in a stimulus condition with chromatic noise, Ѱ_o_ is the threshold estimated in the stimulus condition with no chromatic noise.

**Figure 6:**
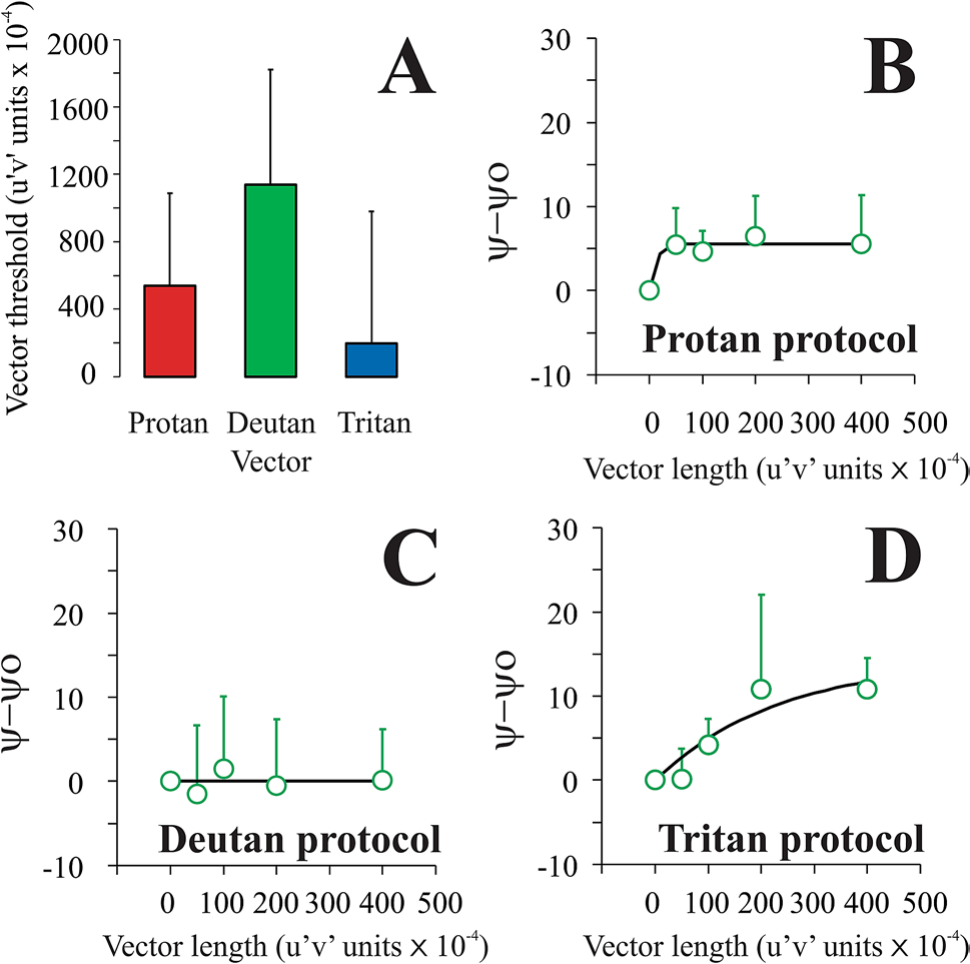
Deutan group results. (**A**) Mean color discrimination thresholds estimated using CAD test for protan (red bar), deutan (green bar), and tritan (blue bar) confusion axes. (**B**) Mean luminance contrast threshold (green circles) as a function of the protan chromatic noise saturation. (**C**) Mean luminance contrast threshold (green circles) as a function of the deutan chromatic noise saturation. (**D**) Mean luminance contrast threshold (green circles) as a function of the tritan chromatic noise saturation. The black curve represents the best-fitted Michaelis–Menten function to the data. Error bars represent the standard deviation of the mean. Ѱ is the threshold estimated in a stimulus condition with chromatic noise, Ѱ_o_ is the threshold estimated in the stimulus condition with no chromatic noise.

Figures 4, 5, and 6 show the mean luminance contrast thresholds as a function of the chromatic noise vector size (saturation) obtained from trichromats, and for subjects with protan and deutan losses, respectively. For trichromats, the results were similar to those observed in Miquilini et al.^5^ for all chromatic noise protocols. The luminance thresholds increased as a function of chromatic noise saturation. We observed a significant effect of the chromatic noise vector size (saturation) on the luminance contrast discrimination (protan chromatic noise protocol: X^2^[4] = 37.75, p = 0.0001; deutan chromatic noise protocol: X^2^[4] = 29.6, p = 0.0001; tritan chromatic noise protocol: X^2^[4] = 25.09, p = 0.0001).

**Figure 7:**
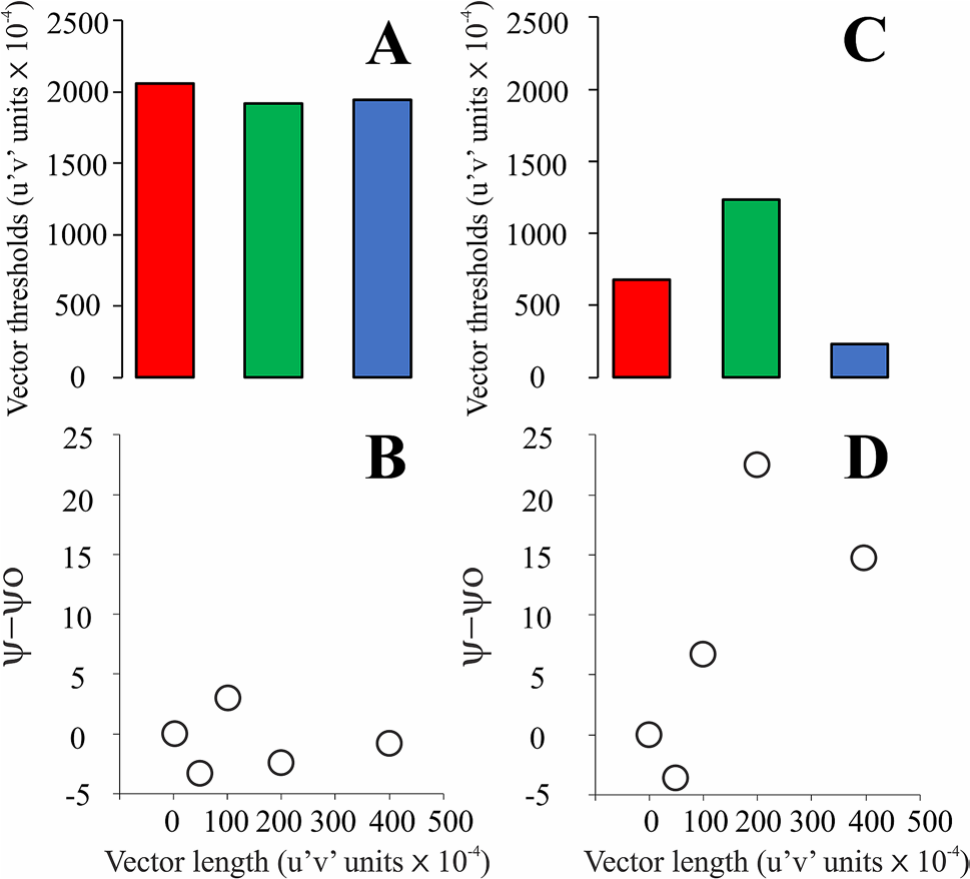
Examples of subjects with color vision losses (observers P4 and D5) with and without the inhibitory influence of the tritan chromatic noise on the luminance discrimination. P4 has poor chromatic discrimination on the tritan (**A**) axis and no significant change on the luminance discrimination (circles) as the saturation of the tritan chromatic noise was increased (**B**), while D5 has better chromatic discrimination on the tritan axis (**C**) compared to the observer P4 and significant increase of the luminance discrimination thresholds as the saturarion of the tritan chromatic noise increased (**D**). Color bars represented the chromatic discrimination on the protan (red bars), deutan (green bars), and tritan (blue bars) axes.

**Figure 8:**
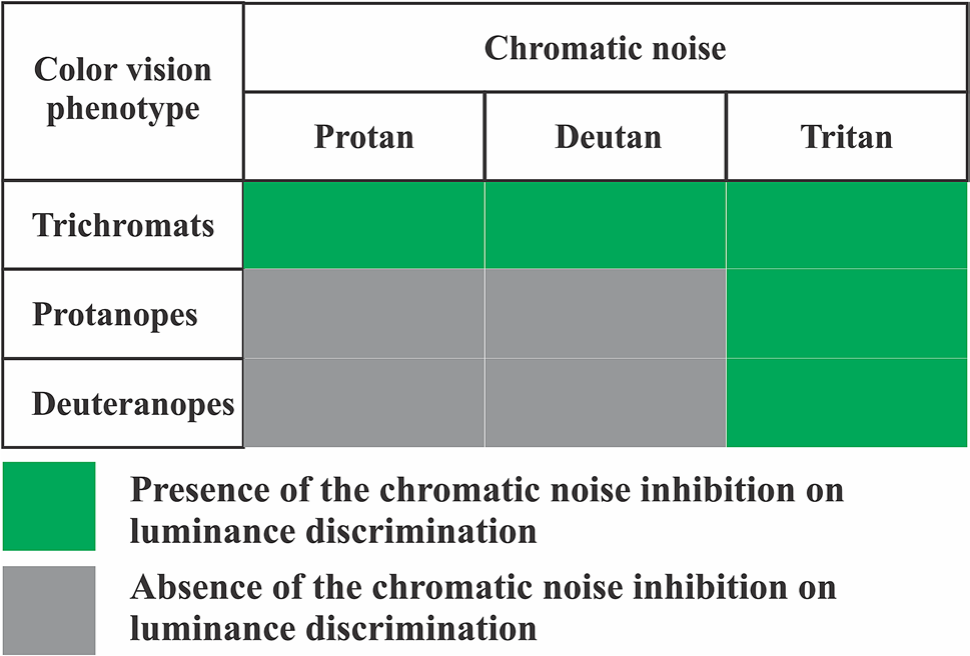
Summary of the results from the evaluation of the influence of chromatic noise experiments on the luminance contrast discrimination. All chromatic noises significatively influenced the threshold discrimination of trichromats, only the tritan chromatic noise significatively influenced the luminance thresholds of the dichromats.

For the participants with color vision deficiencies, the variation in the luminance contrast threshold exhibited a nonsystematic change as a function of the chromatic noise vector size for the protan and deutan chromatic noise protocols. The luminance contrast thresholds increased as a function of chromatic noise saturation in 8 of the 10 subjects in the tritan chromatic noise protocol. Figure 7 shows an example of a participant (observer P4) with no significant inhibitory influence of tritan chromatic noise on luminance discrimination. Furthermore, a subject (observer D5) with a significant inhibitory influence of tritan chromatic noise on luminance discrimination was also observed. The chromatic noise vector did not have a significant effect on the luminance contrast thresholds (protan chromatic noise protocol: X^2^[4] = 7.84, p = 0.1; deutan chromatic noise protocol: X^2^[4] = 3.68, p = 0.45; tritan chromatic noise protocol: X^2^[4] = 5.6, p = 0.23). We expected a significant influence of tritan chromatic noise on the luminance discrimination of protanopes/severe protanomalous. However, there was no influence because 2 protanopes/protanomalous with poor discrimination on the tritan axis increased the variability of the data for the measurements on each chromatic noise vector. For deuteranopes/severe deuteranomalous, there was no significant effect of the chromatic noise vector on the luminance contrast thresholds when the stimulus was comprised of protan and deutan chromatic noise protocols (protan chromatic noise protocol: X^2^[4] = 0.36, p = 0.98; deutan chromatic noise protocol: X^2^[4] = 1.6, p = 0.8). However, a significant effect was found for the tritan chromatic noise protocol (X^2^[4] = 13.92, p = 0.008). Figure 8 summarizes the results of the inhibitory influence of the chromatic noise protocols on luminance discrimination for all the color vision phenotypes studied.

For trichromats, $a$ values estimated from the fits ranged between 14.23–39.71%, 4.83–22.53%, and 4.76–55.33% for the protan, deutan, and tritan chromatic noise protocols, respectively. We found a significant effect of the chromatic noise protocol on the $a$ values (X^2^[2] = 7.091, p = 0.02). $a$ values obtained from the deutan chromatic noise protocol were significantly smaller than those obtained from the protan chromatic noise protocol (p = 0.03). No other multiple comparisons between $a$ values obtained from trichromats displayed significant differences.

For the participants with color vision deficiencies, $a$ values ranged between 1.99–19.76%, − 8.1 to 22.41%, and 1.83–25% for the protan, deutan, and tritan chromatic protocols, respectively. No significant effect of the chromatic noise protocol on the $a$ values was observed (X^2^[2] = 3.2, p = 0.22).

**Figure 9:**
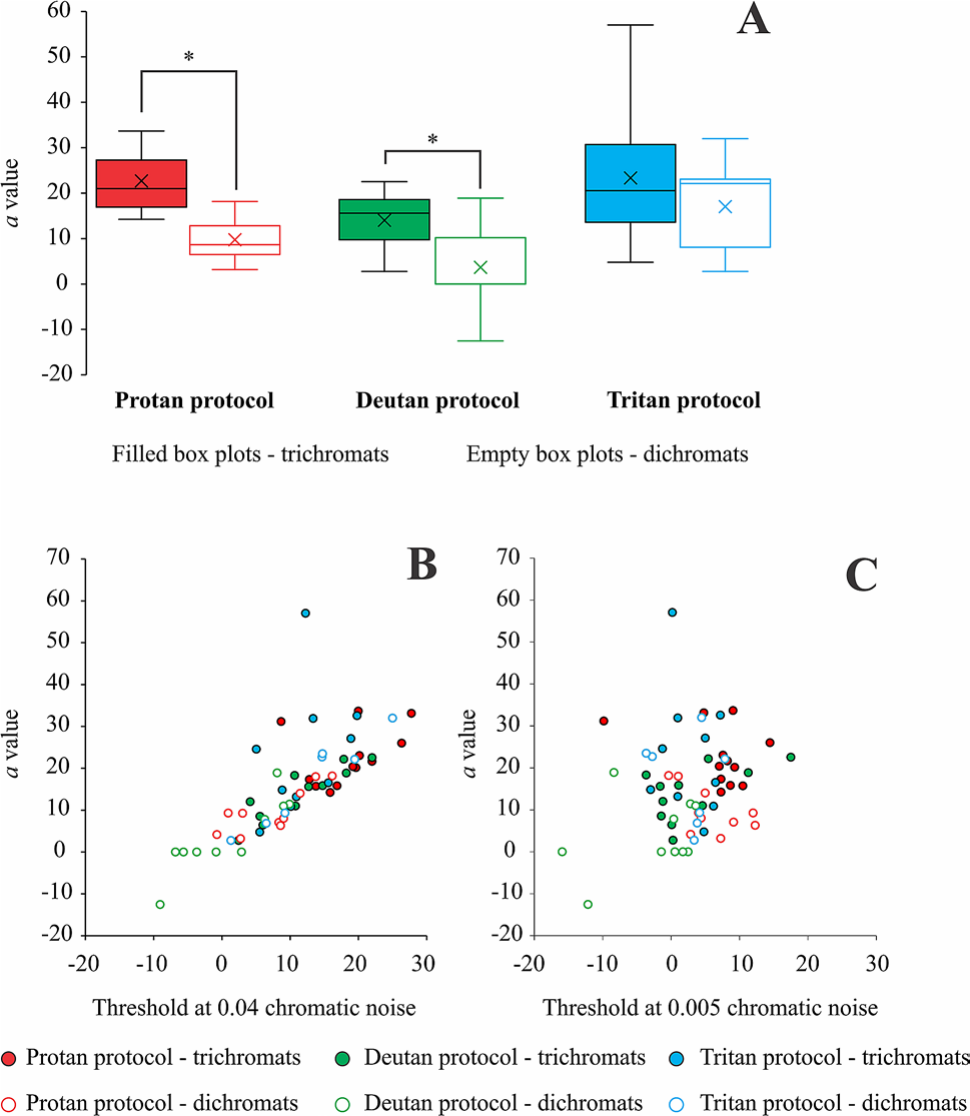
Comparison of the *a* values obtained from the individual fittings of the trichromats and dichromats/severe anomalous trichromats, using the different chromatic noise protocols (**A**). (**B**,**C**) represent the a values as a function of the luminance thresholds obtained in chromatic noise conditions of 0.04 and 0.005 u’v’ units, respectively. Box plots are represented by the interquartile range (box), median (horizontal line inside the box), maximum and minimum values (upper and lower whiskers, respectively), and mean (cross).

A comparison between the results obtained from trichromats and subjects with severe color vision deficits showed a significant effect of the protan and deutan protocols on the $a$ values (protan chromatic noise protocol: U = 8, p = 0.002; deutan chromatic noise protocol: U = 18, p = 0.0076). Trichromats had larger $a$ values considering both chromatic noise protocols. The tritan protocol had no significant effect on the $a$ values obtained from both trichromats and dichromats/anomalous trichromats. Figure 9A shows a comparison of the values obtained from trichromats and subjects with severe color vision deficit. A linear relationship was observed between the luminance thresholds obtained under chromatic noise masking of 0.04 u′v′ units and the *a* values. However, under the chromatic noise condition of 0.005 u′v′, the luminance thresholds were not good predictors of the *a* values (Fig. 9B,C).

## Discussion

This study continues the investigation initiated by Miquilini et al.^5^, which reported an inhibitory effect of chromatic noise on luminance discrimination. In that study, the experimental procedures were performed using normal trichromats, and the chromatic noise was composed of chromaticities with no color confusion line specificity. Their primary aim was to investigate how the presence of chromatic noise would interfere with luminance discrimination. This study has a similar aim, but we focused on three chromatic combinations. We tested whether a selective chromatic noise built with colors close to the color confusion lines of subjects with severe color vision impairments would influence the luminance discrimination of normal trichromats and those of dichromats or severe anomalous trichromats. Although trichromats were far more affected in all three axes by chromatic noise, protanopes/severe protanomalous and deuteranopes/severe deuteranomalous were indistinguishable. Many color vision tests can already predict the deutan vs. protan deficit, but this threshold test results in an equal deficit for both axes.

We used two red-green noises (protan and deutan noises) and one blue-yellow noise (tritan noise) to evaluate the influence of these chromaticities on the luminance discrimination of trichromats and the participants with congenital color vision deficiencies. Many psychophysical studies have investigated the interactions between color and luminance using stimuli containing luminance and color components^1,2,5,15,16^. A facilitative influence of luminance masking on chromatic discrimination and inhibitory influence of chromatic masking on luminance discrimination has been reported. In this study, trichromat participants showed impaired luminance discrimination in all chromatic noise protocols (protan, deutan, and tritan noises). Higher discrimination thresholds were found as the saturation of the colors composing the noise increased. As such, it appears that the noise effectively masked the luminance contrast discrimination because trichromats discriminate all components of chromatic noise. No significant changes in luminance discrimination were observed in subjects identified with severe color vision deficiencies using masking with protan and deutan chromatic noises. We observed that noise did not effectively mask the perceptual task because dichromats or severe anomalous trichromats were not differentially sensitive to the components of chromatic noise. Protan and deutan chromatic noises exerted residual influence on the luminance discrimination of deuteranopes/severe deuteranomalous and protanopes/severe protanomalous, because both color vision deficiencies are for red-green chromatic discrimination. However, the luminance discrimination of these subjects was masked by tritan noise because they have a blue-yellow perceptual dimension.

We found non-independent effects of the protan and deutan protocols on the luminance contrast thresholds of protanopes/protanomalous and deuteranopes/deuteranomalous, which could indicate that both chromatic noise protocols act on one L–M opponent mechanism. We interpreted this as a limitation in separating both color vision phenotypes, but it is a strong indication of the interaction between the information processed by the color- and luminance-opponent pathways. The use of the luminance thresholds under noise conditions of highly saturated chromaticity was a good predictor of the inhibitory influence of chromatic noise on luminance discrimination.

A limitation of this study was the absence of tritanope observers to verify the specificities of the phenotype owing to the inhibitory influence of luminance discrimination. There are few tritan congenital subjects, and it can be challenging to identify^17^. As an approximation, it seems relevant that some of the participants with severe color vision deficiency who had very poor discrimination on the tritan axis in the CAD evaluation had no inhibitory influence of tritan chromatic noise on luminance discrimination, as would be expected for tritanopes. More subjects with poor discrimination on the tritan axis are required to confirm this hypothesis. A recent study found worse color discrimination on the tritan axis for deutan participants, due to a higher impact on the yellow region of the color space^18^. New experiments should also be carried out to clarify the effect of contrast polarity (luminance decrements or increments) on the luminance discrimination in the presence of chromatic noise masking.

The protocols discussed in this study enable new visual tests to be created that simultaneously evaluate color and luminance vision because the observer’s task is to discriminate luminance contrast, and the effect of chromatic noise is dependent on the color vision phenotype. A comparison between luminance contrast discrimination obtained under no chromatic and high-saturated chromatic noise conditions could be used to initially compose this double-duty test. As several congenital and acquired diseases can affect color and luminance vision, our results encourage the use of the present protocols in clinical practice.

## Data Availability

The datasets generated during and/or analyzed during the current study are available from the corresponding author on reasonable request.
